# Analysis of Adverse Effects of COVID-19 Vaccines in Spain following Booster Dose

**DOI:** 10.3390/vaccines10091397

**Published:** 2022-08-25

**Authors:** Esther Ríos, Sara Medrano, Mercedes Martínez, Consuelo Novella, Esther Marcos, Jose J. Fernández, Alberto Delgado-Iribarren, Esther Culebras

**Affiliations:** 1 Department of Medicine, Facultad de Medicina, Universidad Complutense Madrid, 28040 Madrid, Spain; 2 Department of Clinical Microbiology, IdISSC, Hospital Clínico San Carlos, 28040 Madrid, Spain; 3 Departamento de Biología del Servicio de Criminalística de la Guardia Civil, 28003 Madrid, Spain

**Keywords:** COVID-19 vaccines, third dose, heterologous prime–boost, secondary effects, serious event

## Abstract

The present study evaluates the adverse effects of three vaccines: AstraZeneca (Vaxzevria), Pfizer/BioNTech (Comirnaty) and Moderna (Spikevax) according to the dose. From 733 participants collected, the vaccine schedule was as follows: 330 (45%) received a double dose of the AstraZeneca vaccine, 382 (52.1%) received a double dose of Pfizer, 18 (2.5%) received a heterologous prime boost and 3 (0.4%) received a single dose. Pfizer and Moderna vaccines were administered as a third dose in 70 and 121 individuals, respectively. Local and systemic reactions observed in the three vaccines were mild to moderate in severity. Only one AstraZeneca recipient (0.3%) presented a serious adverse effect: blurred vision. Adverse events were more frequent after the first dose of AstraZeneca and after the second dose of Pfizer. As the third dose, Moderna causes more adverse effects than Pfizer regardless of the type of vaccine previously administered, whereas the reactogenicity of a third dose of Pfizer is slightly higher in the group previously vaccinated with Pfizer than in that group with AstraZeneca. In short, secondary effects of the third dose of COVID-19 vaccines were similar to those after dose 2, but their frequency depends on the type of vaccine and the combinations of vaccines.

## 1. Introduction

The “Severe Acute Respiratory Syndrome Coronavirus 2 (SARS-CoV-2)” disease has caused a threatening pandemic globally (COVID-19) [1]. According to the World Health Organization (WHO) as of 8 July 2022, globally, there have been 551,226,298 confirmed cases of COVID-19, including 6,345,595 deaths [2]. Vaccines provide immunogenicity against a SARS-CoV-2 infection. Several vaccines for COVID-19, have been developed. Among the vaccines available in Spain, two of them, Pfizer/BioNTech (Comirnaty) and Moderna (Spikevax), are messenger RNA (mRNA) vaccines. Other vaccines, such as AstraZeneca (Vaxzevria), are made using primate adenovirus vectors [3]. The three vaccines were authorized for use in Europe by the European Medicines Agency (EMA).

In Spain, vaccinations were carried out according to an order of priority based on the risk of serious disease and the risk of exposure fundamentally. Pfizer/BioNTech was the first administered vaccine. Later, Moderna and AstraZeneca were progressively introduced [4].

According to the vaccine instructions and clinical trial design, two doses are required to ensure adequate protection for most of these vaccines, and the prime–boost interval ranged from 21 days to 3 months [5]. The two doses could be the same (homologous vaccination) or different (heterologous vaccination). The European Medicines Agency (EMA) and the European Center for Disease Prevention and Control (ECDC) endorse heterologous vaccination; that is, they “mix and match” different COVID-19 vaccines in both primary and booster vaccination [6].

COVID-19 vaccines have been shown to produce mild-to-moderate adverse effects that go away within a few days on their own [7,8,9,10,11,12,13,14,15,16,17]. These reactions are reported to be less frequent in the Pfizer vaccine compared to the Moderna COVID-19 vaccine [1]. Considering dose, adverse effects have been described to be greater in the 1st dose of AstraZeneca [9,10] and the 2nd dose of Pfizer [13,14,16]. Local and systemic reactogenicity events from the third dose of COVID-19 vaccines have been similar to those after dose 2 [18].

This is an observational descriptive study whose general aim was to analyze and compare the adverse effects of three vaccines used in Spain: AstraZeneca, Pfizer and Moderna according to dose, vaccine or combination of vaccines. As previously described [1], the Moderna vaccine causes more reactogenicity than Pfizer; therefore, we hypothesized that a third dose of Moderna would also produce more adverse effects than Pfizer. Moreover, we evaluated whether the influence of the third dose of either vaccine could have been based on previous vaccination strategies.

## 2. Materials and Methods

### 2.1. Population Study

In this descriptive observational study, we collected and evaluated the secondary adverse events of three vaccines used in Spain: AstraZeneca, Pfizer and Moderna according to the administered dose. The participants were recruited through different routes: Those vaccinated with Pfizer and Moderna were recruited among the health workers of the Hospital Clínico San Carlos (HCSC) and through the HCSC retirees’ association. Those vaccinated with AstraZeneca came from different public administrations, mainly from the Spanish Civil Guard (Guardia Civil) and teachers of different levels (primary, secondary and University). In addition, this last group included relatives aged over 65 years of hospital workers.

The different vaccines were given at different times. Dosing was performed as follows: Pfizer and Moderna vaccines was given 21 and 28 days apart, respectively. The second dose of the AstraZeneca vaccine was given 8–12 weeks after the first dose regardless of whether that second dose was the same vaccine or a different one. The third dose was only given to people who had received the second dose at least 5 months ago.

### 2.2. Procedures

The enrolled participants provided written informed consent and completed a questionnaire, indicating age, sex, type of vaccine received, number of doses of the vaccine taken and secondary effects after each dose of vaccine (injection site pain, headache, fever, fatigue, dizziness and adenopathy). Respondents could also add other symptoms and if they had previously been infected by SARS-CoV-2.

Secondary effects were recorded by the participants themselves. Any unusual symptoms detected after the administration of each of the doses were considered to be a secondary effect of the vaccine. Other information was also included, such as underlying diseases and medication habitually taken (if any). In addition, each participant’s telephone number was noted for future contacts.

Contact was maintained with all participants to monitor their response to vaccination. Two copies of the questionnaire were initially given to all of them, and they were asked to fill them out twenty days after the administration of each of the first two doses. When it was decided that a third dose was appropriate, they were given an extra copy of the questionnaire to complete.

Ethical approval for use of the data from the questionnaires for research purposes was obtained from the Ethics Committee of Hospital Clínico San Carlos, Madrid, Spain (References: 21/071-E and 21/193-E).

### 2.3. Statistical Analysis

Categorical variables were analyzed using frequencies and percentages and compared by Fisher´s exact test using 2 × 2 contingency tables. Two-tailed *p*-values < 0.05 were considered statistically significant.

All statistical analysis was conducted using GraphPad Prism v.5.01.

## 3. Results

### 3.1. Participants

Between January 2021 and March 2022, a total of 733 individuals were enrolled. The age of the vaccinated people varies between 24 and 83 years old. Of the participants, 445 (60%) were women and 296 were men (40%). The secondary effects observed with the different vaccines and regimens were similar regardless of sex or age.

### 3.2. Vaccine Schedule

Table 1 shows the distribution of administered vaccines according to the dose. All participants had received at least two doses (homologous or heterologous prime–boost) except for three participants. Of these three participants, two had been infected by SARS-CoV-2 after the first dose, and one of them had received only one dose of AstraZeneca because of a familiar history of thrombus risk.

Homologous vaccinations were administered in a total of 712 people, 382 were vaccinated with two doses of Pfizer and 330 were vaccinated with a double dose of AstraZeneca. Among the 712 participants, 183 also received a third dose (66 Pfizer and 117 Moderna).

The rest of the participants received a heterologous vaccination. Of the 18 individuals previously primed with AstraZeneca vaccine, 14 received an alternative vaccine (Pfizer) as their second dose, and four people received Moderna as their third because they had been infected by SARS-CoV-2 after the first dose (Table 1).

Overall, six participants were infected by SARS-CoV-2 after the first dose of the AstraZeneca vaccine, and one person reported that they had tested positive for COVID-19 after the first dose of the Pfizer vaccine.

### 3.3. Adverse Effects after Homologous Vaccination with Pfizer

**Figure 1:**
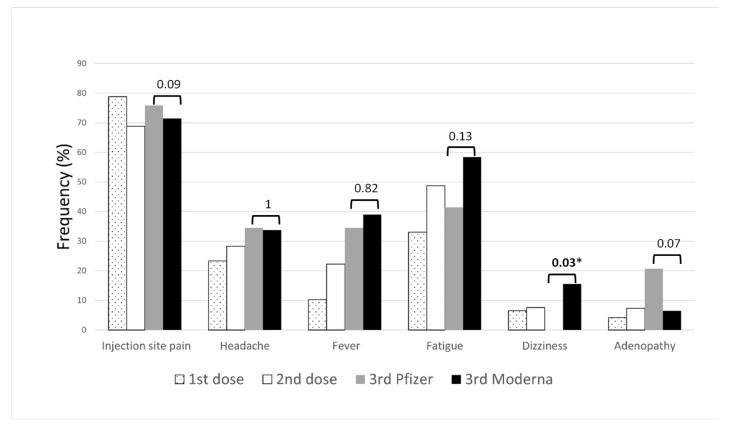
Adverse effects among people vaccinated with a double dose of Pfizer. 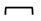 bars represent *p*-values. * *p*-values < 0.05.

Adverse effects among people vaccinated with a double dose of Pfizer are shown in Figure 1. The most frequent adverse effect was pain at the injection site (78.8% 1st dose, 68.85% 2nd dose), followed by fatigue (32.98% 1st dose, 48.69% 2nd dose). Headache, fever, adenopathy and dizziness were also reported. After the second dose of the Pfizer vaccine, many more people had more secondary effects than after the first dose except injection site pain. Additional secondary effects not included in the questionnaire were detected in 86 (22.51%) people vaccinated with Pfizer: itching and redness at the injection site, muscle pain, joint pain, diarrhea, nausea, vomiting, chills, paresthesia, arrhythmia, lethargy (lack of energy), itching, erythema multiforme, menstrual alterations, sweating, cough, sore throat, sore ear and vertigo. In total, 65 (17.02%) people presented no adverse effects after the first dose and 73 (19.1%) presented no adverse effects after the second dose.

Out of 106 people vaccinated with a double dose of Pfizer, 29 received a third dose of Pfizer and 77 received a third dose of the Moderna vaccine. Secondary effects after the third dose are shown in Figure 1. Pain at the injection site was again the most common adverse effect for both Pfizer and Moderna vaccines (75.86% and 71.43%, respectively). Fever, fatigue and dizziness were more frequent after the third dose of Moderna than those of Pfizer. However, pain at the injection site, headache and adenopathies were more reported by the Pfizer than Moderna recipients.

### 3.4. Adverse Effects after Homologous Vaccination with AstraZeneca

**Figure 2:**
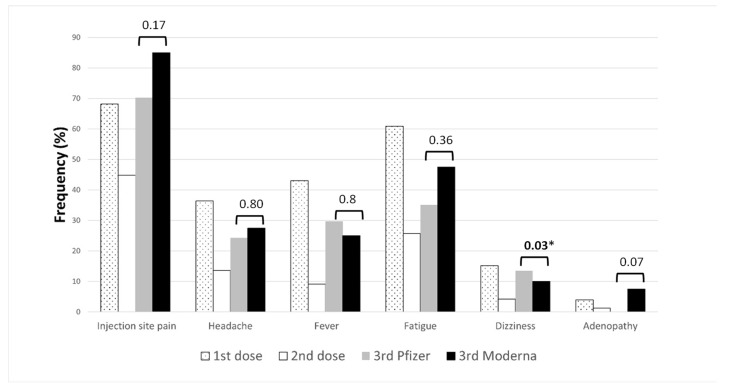
Adverse effects among people vaccinated with a double dose of AstraZeneca. 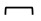 bars represent *p*-values. * *p*-values < 0.05.

Adverse effects among people vaccinated with a double dose of AstraZeneca are shown in Figure 2. The most frequent adverse reaction was injection site pain; among 330 participants, 225 (68.18%) had this effect after the first dose and 157 (44.85%) had this effect after the second dose. After the second dose, people reported fewer secondary effects than after the first dose. Additional secondary effects not included in the questionnaire were described by 52 (15.76%) people vaccinated with AstraZeneca: swelling and redness at the injection site, joint and muscle pain, nausea, vomiting, menstrual alterations, abdominal pain, muscle spasms, pain in legs or arms, itching, rash and urticaria, diarrhea, decreased appetite, sleepiness, lethargy, blurred vision, relaxation, hearing loss, ear pain, flulike symptoms such as a sore throat and cough. In total, 66 (20%) people presented no adverse effects after the first dose and 144 (43.64%) presented no adverse effects after the second dose.

Out of 77 people vaccinated with a double dose of AstraZeneca, 37 received a third dose of Pfizer and 40 Moderna vaccines. The secondary effects after the third dose are presented in Figure 2. In general, the third dose of Moderna caused secondary effects more often than the third dose of Pfizer, except fever and dizziness. The most prevalent adverse effect for both vaccines was injection site pain (70.27% Pfizer vs. 85% Moderna). Among 37 people vaccinated with a 3rd dose of Pfizer, 3 (7.5%) had adenopathies; however, no person vaccinated with the 3rd dose of Moderna reported this effect.

### 3.5. Reactogenicity of Pfizer Booster in AstraZeneca-Primed Participants

**Figure 3:**
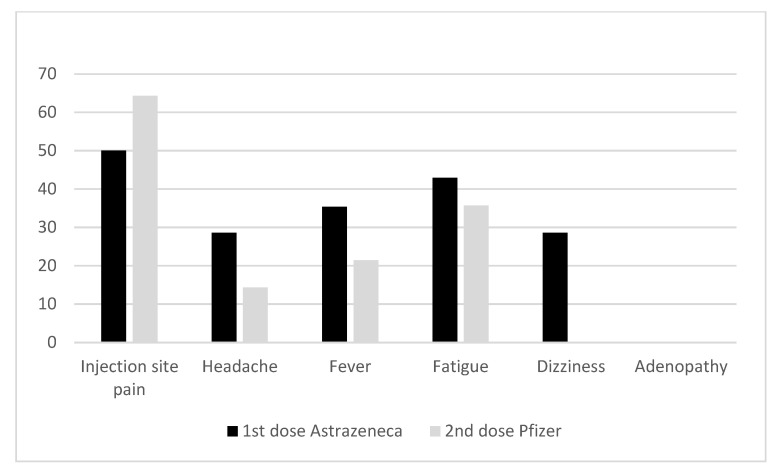
Adverse effects among participants with heterologous vaccination.

The frequencies of adverse events in the 14 participants with heterologous vaccination are shown in Figure 3, with injection site pain (64.3%), fatigue (35.7%) and fever (21.4%) being the most commonly reported adverse events. No adenopathy or dizziness were observed. Among the 14 individuals, four were vaccinated with a third dose of Pfizer (Table 1), reporting similar adverse events than after the 2nd dose.

### 3.6. Statistical Analysis

Table 2 includes the statistical values for all comparisons in the study.

Statistical analysis of secondary effects of the first and second doses of each vaccine showed that all events were significantly reduced after the administration of the second dose of AstraZeneca. However, this decrease was not so obvious when the vaccine used was Pfizer.

When a comparison between vaccines was made, it was observed that AstraZeneca produced more adverse reactions than Pfizer with the first dose, but the opposite occurred with the second one. The differences were statistically significant for all adverse effects except for adenopathy in dose 1 and dizziness in dose 2.

The differences between secondary effects produced by the Pfizer or Moderna booster vaccination were not statistically significant neither in the case of two previous doses of AstraZeneca nor in that of Pfizer. There was only one exception of dizziness when a double dose of Pfizer was administered.

## 4. Discussion

COVID-19 vaccination helps protect people from getting COVID-19. Like any vaccine, COVID-19 vaccines can cause secondary effects, most of which are mild or moderate and go away within a few days on their own. Vaccines are continually monitored to detect adverse events [7].

In the present study, we evaluated the secondary effects of the Pfizer, AstraZeneca and Moderna vaccines. Local and systemic reactions observed were mild to moderate in severity and comparable to those previously reported in participants after vaccination with AstraZeneca [8,9,10], Pfizer [11,12,13,14,15,16] or Moderna [1,17]. Only one AstraZeneca recipient (0.3%) presented a serious adverse effect: blurred vision. Neurological symptoms after AstraZeneca vaccination, such as blurred vision, have been published by the EMA [19]. Healthcare professionals should be alert to the signs and symptoms of thromboembolism and/or thrombocytopenia.

The incidence of rare adverse events was 0.5%; four participants reported hearing-related symptoms (hearing loss, ear pain, vertigo) after receiving any of the vaccines. Otologic manifestations after COVID-19 vaccination have been mentioned in the literature [20,21]. However, further studies should be needed to determine if the reported otological alterations were vaccine-induced or due to some underlying pathology or previous medication interactions.

Comparing the frequencies of adverse effects of the three vaccines in our study, the highest rate of fever was found in individuals primed with AstraZeneca (43.03%), as previously described in a systematic review by Nan-chang et al. [5].

Injection site pain and fatigue were the most frequently reported secondary effects of the three vaccines studied. The rates of adverse events following the AstraZeneca vaccine were comparable to those observed in previous clinical trials [9] where injection site pain and fatigue were the most frequent. Nevertheless, the frequencies of each secondary effects were higher than expected from the community studies [14]. AEMPS published in the 11th pharmacovigilance report that feverishness and headache were the most common adverse effects among AstraZeneca recipients until December 2021, whereas the frequency of injection site pain and fatigue was 10% for each effect [22].

Similarly, the frequencies of adverse effects of Pfizer were more similar to clinical trials [13] than to later published studies [10,14,15,16]. We found that the most common events after both doses of Pfizer were pain at the injection site (78.8% 1st dose, 68.85% 2nd dose), which was followed by fatigue (32.98% 1st dose, 48.69% 2nd dose). These two secondary effects were the most prevalent in clinical trials [13]. Notwithstanding, our percentages were higher than those previously observed [10,14,15,16]. For instance, injection site pain was reported in 63.3% and 57.1% of recipients after the 1st and 2nd dose, respectively, in a study carried out in Poland.

Regarding the groups with homologous vaccine schedules, our findings showed that adverse effects were more common after the prime dose in the AstraZeneca group and after the boost dose in the Pfizer group. In the case of the Pfizer vaccine, systemic events have been observed more often after dose 2 than dose 1 [10,13,14,16]. However, people receiving the 2nd dose of AstraZeneca have reported secondary effects less frequently than after receiving the 1st dose [8,9].

Regarding the 3rd doses received, our results are comparable with those previously published in a clinical trial where adverse reactions after dose 3 were similar to those after dose 2 [18]. In general, Moderna caused secondary effects more often than Pfizer. Adverse events are reported to be lower in the Pfizer vaccine compared to the Moderna vaccine [1]. This fact could be explained because the two vaccines have certain differences in their properties and actions on the body. A third dose of Pfizer is given in a dosage of 30 μg, whereas the Moderna vaccine is given in a 50 μg dosage [1].

Moreover, the present study demonstrates that the 3rd dose of Pfizer causes slightly higher reactogenicity rates in the group previously vaccinated with Pfizer than in that group with AstraZeneca. For example, fever was reported by 10 (34.48%) of 29 recipients of the Pfizer group compared to 11 (29.73%) of 77 recipients of the AstraZeneca group. Similar increases were observed for injection site pain, fatigue, headache and adenopathy.

In general, adverse effects of Pfizer were more common after dose 3 than dose 2. Accurately, the percentage of adenopathy was higher in the participants who had received a booster dose (third dose) than in those with two doses of Pfizer (20.69% vs. 7.3%). A high proportion of adenopathy has been previously described after the administration of the 3rd dose of Pfizer in clinical trials [16]. Our results differ from those observed in a clinical trial with participants who had received three doses of Pfizer [18], local reactions were reported more frequently after dose 3 than dose 2, whereas systemic reactions were less frequent after dose 3 than dose 2.

Finally, in the present study, we also evaluated the adverse events of Pfizer booster in AstraZeneca-primed participants, being the frequencies similar or even lower than those that had received a homologous Pfizer scheme and higher than homologous AstraZeneca vaccination. Several studies revised by Sapkota et al. [23] had described greater systemic reactogenicity with heterologous boosters than their homologous counterparts. Therefore, our results should be carefully interpreted and compared with published literature because characteristics between studies could be different.

A limitation of the study may be the sample size, since it had not been adjusted to any previous calculation. The data were retrieved only through the questionnaire that the participants voluntarily gave us.

In conclusion, as the third dose, Moderna causes more adverse effects than Pfizer regardless of the type of vaccine previously administered. Nevertheless, the reactogenicity of Pfizer, as the third dose, is slightly higher in the group previously vaccinated with Pfizer than in that group with AstraZeneca.

## Figures and Tables

**Table 1:** Vaccine schedule of the 733 participants.

1st Dose	2nd Dose	3rd Dose	No. of Participants
PZ	PZ	PZ	29
PZ	PZ	Mo	77
PZ	PZ	-	276
PZ	-	-	1
AZ	AZ	PZ	37
AZ	AZ	Mo	40
AZ	AZ	-	253
AZ	PZ	-	10
AZ	PZ	PZ	4
AZ	-	-	2
Az	-	Mo	4

AZ: Vaxzevria (AstraZeneca); PZ: Comirnaty (BioNTech/Pfizer) y Mo: Spikevax (Moderna).

**Table 2:** Statistical values for the comparison of categorical variables in the conditions analyzed.

	AZ/AZ/PF vs. AZ/AZ/Mo	PF/PF/PF vs. PF/PF/Mo	1st Dose AZ vs. 1st Dose PF	2nd Dose AZ vs. 2nd Dose PF	1st Dose AZ vs. 2nd Dose AZ	1st Dose PF vs. 2nd Dose PF
**Injection site pain**						
*p*-value	0.1699	0.0879	**0.0015**	**<0.0001**	** < 0.0001 **	** 0.0023 **
95% CI	0.1363 to 1.276	0.4699 to 3.363	0.4115 to 0.8081	0.2708 to 0.5000	1.918 to 3.620	1.212 to 2.332
**Headache**						
*p*-value	0.7995	1	** 0.0001 **	** <0.0001 **	** <0.0001 **	0.1365
95% CI	0.3047 to 2.357	0.4198 to 2.539	1.357 to 2.608	0.2725 to 0.5889	2.459 to 5.325	0.5566 to 1.067
**Fever**						
*p*-value	0.7985	0.8227	** <0.0001 **	** <0.0001 **	** <0.0001 **	** <0.0001 **
95% CI	0.4647 to 3.467	0.3378 to 2.013	4.468 to 9.876	0.2236 to 0.5459	4.893 to 11.66	0.2637 to 0.5985
**Fatigue**						
*p*-value	0.3556	0.1312	** <0.0001 **	** <0.0001 **	** <0.0001 **	** <0.0001 **
95% CI	0.2393 to 1.498	0.2109 to 1.195	2.328 to 4.305	0.2660 to 0.5025	3.224 to 6.256	0.3870 to 0.6951
**Dizziness**						
*p*-value	0.7306	** 0.0337 **	** 0.0002 **	0.0817	** <0.0001 **	0.6723
95% CI	0.3472 to 5.695	0.005083 to 1.552	1.539 to 4.226	0.2799 to 1.039	2.181 to 7.450	0.4894 to 1.485
**Adenopathy**						
*p*-value	0.2413	0.0666	1	** <0.0001 **	** 0.0462 **	0.0866
95% CI	0.007125 to 2.864	1.048 to 13.47	0.4443 to 1.981	0.05382 to 0.4472	1.078 to 10.36	0.2939 to 1.039
**Others**						
*p*-value	0.1891	0.6708	** 0.0212 **	** <0.0001 **	** <0.0001 **	** 0.0088 **
95% CI	0.5031 to 44.42	0.04863 to 3.673	1.087 to 2.709	0.07204 to 0.3022	3.076 to 13.19	0.3529 to 0.8494

Statistically significant values appeared in bold and gray shaded. AZ: Vaxzevria (AstraZeneca); PZ: Comirnaty (BioNTech/Pfizer) y Mo: Spikevax (Moderna).
